# Gene Therapy for Cystic Fibrosis: Progress and Challenges of Genome Editing

**DOI:** 10.3390/ijms21113903

**Published:** 2020-05-30

**Authors:** Giulia Maule, Daniele Arosio, Anna Cereseto

**Affiliations:** 1 Department of Cellular Computational Integrative Biology (CIBIO), University of Trento, 38123 Trento, Italy; giulia.maule@unitn.it; 2 National Council of Research, CNR, 38123 Trento, Italy; daniele.arosio@cnr.it

**Keywords:** programmable nucleases, CRISPR-Cas, genome editing

## Abstract

Since the early days of its conceptualization and application, human gene transfer held the promise of a permanent solution to genetic diseases including cystic fibrosis (CF). This field went through alternated periods of enthusiasm and distrust. The development of refined technologies allowing site specific modification with programmable nucleases highly revived the gene therapy field. CRISPR nucleases and derived technologies tremendously facilitate genome manipulation offering diversified strategies to reverse mutations. Here we discuss the advancement of gene therapy, from therapeutic nucleic acids to genome editing techniques, designed to reverse genetic defects in CF. We provide a roadmap through technologies and strategies tailored to correct different types of mutations in the cystic fibrosis transmembrane regulator (*CFTR)* gene, and their applications for the development of experimental models valuable for the advancement of CF therapies.

## 1. Introduction

Cystic fibrosis (CF) is an autosomal recessive disease caused by mutations in the cystic fibrosis transmembrane regulator (*CFTR*) gene. *CFTR* encodes for a cAMP regulated chloride channel located in the apical membrane of epithelial cells that catalyze the passage of small ions through the membrane. Dysregulation of this mechanism causes an impairment of salt and fluid homeostasis that results in multiorgan dysfunctions and ultimately mortality from respiratory failure. For more than seven decades, therapies for CF were limited to the treatment of the symptoms rather than addressing the origin of the disease, the altered *CFTR* gene. The mutations causing CF are 352 distributed along the *CFTR* gene, including exonic and intronic regions [1]. Potentiators and correctors are small molecules targeting the large majority (90%) of CF-causing mutations which act by improving trafficking and processing of the mutated CFTR produced. Yet, 10% of disease-causing variants are not responsive to any available small-molecule therapies. Unlike CFTR modulators working on selected mutations, genetic interventions could in principle target any alteration at the basis of the disease and, more importantly, provide a permanent solution to the disease. Indeed, since the first conceptualization of gene therapy for the treatment of genetic diseases by Friedman and Roblin [2], recessive monogenic diseases like CF are optimal targets for therapy by genetic complementation. 

Despite the fact that CF is a multiorgan disease, improving respiratory manifestations by genetic intervention in lungs could result in a significant amelioration in the patient’s quality of life and decreased mortality. Soon after the identification and isolation of the CFTR gene reported in three back-to-back papers in 1989 [3,4,5], a growing number of gene therapy strategies and clinical trials for CF were attempted bringing to light the major challenges of these therapeutic approaches [6,7,8]. Natural barriers to gene therapy are mainly represented by mucus, immune responses to viral vectors or non-viral delivery tools and lack of clearly defined cellular targets. Moreover, the airway epithelium is constantly renewing thus preventing durability of the corrected cells. The methods for CFTR delivery, either viral or non-viral that were developed and tested in pre-clinical and clinical trials, have been widely covered in the literature; we refer readers to recent reviews on this topic [9,10,11,12]. Clinical trials in CF patients were initiated in 1993 testing the delivery of a wild-type copy of the *CFTR* gene in both nasal and bronchial airway epithelium [13]. More than 20 clinical trials were attempted without achieving an effective cure; only limited clinical benefits were observed including the stabilization of lung function in patients who received at least nine doses of *CFTR* cDNA delivered by cationic liposomes over a 12-month period [14]. 

The recent advancement of genetic engineering in living cells with the development of more efficacious biotechnology tools greatly renewed the enthusiasm for gene therapy, as evidenced by the steep increase of articles in scientific and popular publications and calling for continued investment in the field. Genome manipulation has always been a crucial procedure in biological research and for the development of therapies aiming at endogenous gene correction. Soon after the discovery of DNA and its structure it became clear that a key step to modify the genome is the generation of DNA double strand breaks (DSB) at the target sites [15,16]. As a result of DSB, cells can respond with two different DNA repair mechanisms to prevent cell death: non-homologous end joining (NHEJ) or homology-directed repair (HDR) [17]. NHEJ generates insertion and deletion of variable lengths (indels) that are hardly predictable even with the most recent computational tools when induced by genome editing nucleases [18,19,20]. This repair pathway is mainly used to knockout or knock-in at preferred genomic sites. Instead, HDR allows introducing desired editing in the target sequence. HDR-specific donor templates are introduced by recombination at sites having sufficient sequence homology [17]. Nevertheless, HDR is not broadly exploited to manipulate the genome due to low frequency an efficient process in mammalian cells, generating at best one modified cell out of a million [21,22]. 

A turning point in the history of genome editing is represented by rare-cutting meganucleases (e.g., I-SceI) and their use to enhance gene substitution with donor sequences [16,23], leading to directed and increased HDR at sites of enzyme cleavage. The I-SceI results triggered the search for programmable nucleases which was initially directed to engineer natural meganucleases into enzymes cleaving alternative sites [24]. The field then moved to artificial enzymes, Zinc Finger Nucleases (ZFN) and transcription activator-like effector nucleases (TALEN), which highly facilitated targeted nuclease cleavages [25,26] with successful development of ZFN towards experimental clinic [27,28]; yet the complex and cumbersome protein engineering behind the production of these tools severely limited their widespread acquisition for research use or gene therapy. These limitations were surpassed by the subsequent discovery and development of CRISPR-Cas nucleases, biotechnology tools derived from natural nucleases found in the immune defenses of microbes. This technology literally propelled the gene therapy field, generating a steep increase of novel strategies to reverse genetic aberrations. The supremacy of CRISPR-Cas in genome editing derives from its simplicity in target design, the high specificity obtained with the development of high-fidelity variants, the suitability to multiplexing use and the low cost [29,30,31,32,33,34]. Thanks to the versatility of both protein and guide-RNA (gRNA) modules, a variety of CRISPR derived tools were developed expanding the choices of genome modification strategies. The fusion of Cas with deaminase domains allowed the development of base editors for the modification of single nucleotides [35], while fusion with a reverse transcriptase and a particular extended gRNA allowed the production of prime editing [36], as an alternative to HDR. 

**Figure 1:**
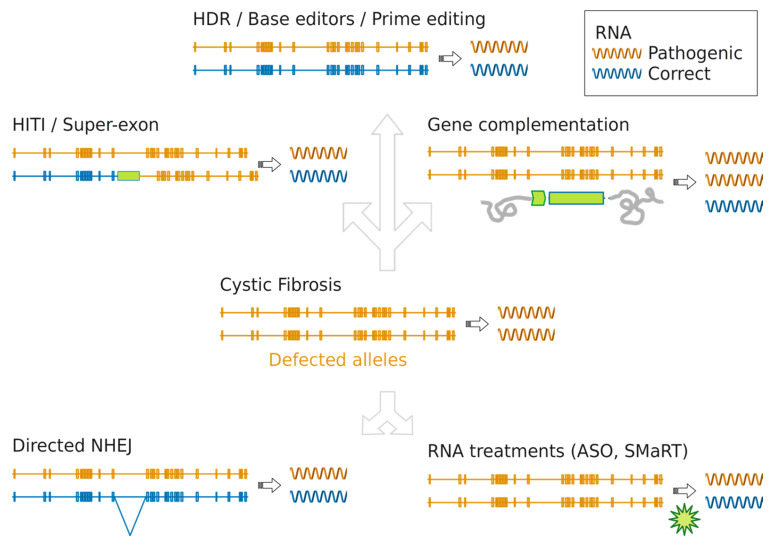
Gene therapy strategies to restore CFTR function in CF. CFTR genes containing any mutation causing cystic fibrosis are colored in orange, genes bearing the correct sequence in blue. Elongated bars indicate the 27 exons distributed over the entire length (188702 bp) of CFTR gene. Striped right arrows indicate the transcription process. In the center panel a typical CF patient condition is depicted; a genetic defect is present on each allele preventing transcription of suitable amount of mRNA molecules with correct sequence. In the gene complementation panel, green arrow indicates the exogenous promoter randomly integrated into the cell genome (grey lines) and able to produce mRNA with correct sequence (blue) in amount sufficient to restore CFTR function. Genome editing strategies able to correct at least a copy of the CFTR gene at the endogenous locus. Graphical representation for the HITI and super-exon strategies: targeted gene correction restores specifically the production of correct mRNA molecules (always in blue). Directed NHEJ is also able to reestablish production of correct mRNA molecules for certain splicing defects thanks to small changes introduced in specific intron regions. RNA treatments intervene (green arrow) during the transcription process to rectify correct mRNA molecules production.

Genome editing has entered the clinic for the treatment of hematologic diseases and cancer by ex-vivo delivery and for genetic blindness by in-vivo delivery through adeno-associated viral vectors [37]. The fast developments of CRISPR technology and the early encouraging pre-clinical and clinical results suggest an upcoming larger use of this technology for the treatment, in-vivo or ex-vivo, of numerous genetic diseases, including CF. Several reports demonstrating the efficacy of genome editing in a wide number of CF models holds the promise of its translation to a clinical use. Here we review the gene therapy strategies developed to either compensate or repair aberration in the *CFTR* locus (schematized and summarized in Figure 1 and Table 1). We provide a roadmap through nucleic acids treatments and genome manipulations tailored to the type of CF mutations and discuss potential new ways to correct the *CFTR* locus.

## 2. Gene Complementation

The monogenic and recessive nature of CF makes this disease an optimal target for gene complementation strategies [2]. The delivery of a wild type copy of the *CFTR* cDNA represents an attractive therapeutic approach suitable for any type of CF mutations [38]. Several pre-clinical studies and clinical trials have been performed testing a variety of delivery tools to restore *CFTR* expression in target cells [9,10,11,12]. Nonetheless, gene complementation is not necessarily the best therapeutic choice. First, the non-physiologic expression of *CFTR* driven by a non-endogenous promoter, most frequently of viral origin. Second, the loss of the *CFTR* transgene generated either by the episomal nature of commonly used viral or non-viral vectors (Adenovirus/Adeno Associated viruses or lipidic complexes) or by the turnover of the airway epithelium. A stable integration of the *CFTR* transgene can be obtained through retroviral vectors and may potentially produce continuous expression by targeting staminal cells in the airway epithelium. Nevertheless, beyond difficulties in reaching the proper target cells in the epithelium, the use of retroviral vectors has been limited by the concern raised by insertional mutagenesis occasionally reported in gene therapy clinical trials [39].

## 3. Targeted Integration

With the recent development of powerful tools to manipulate the genome, new perspectives are opened in gene therapy to restore the correct CFTR expression by engineering the *CFTR* locus thus permitting physiologic expression of the ion channel. Programmable nucleases offer the opportunity to precisely target integration of DNA sequences at their cleavage sites [40,41]. This represents a true advancement relative to conventional integration mediated by retroviral vectors associated with occasional insertional mutagenesis, epigenetic silencing and the altered expression levels driven by an exogenous promoter [39,42,43]. Homology-independent targeted integration (HITI) uses CRISPR-Cas mediated cleavages, both on target and incoming DNA, to catalyze the integration of DNA sequences at specific genomic sites and in the correct orientation [41]. HITI integration does not depend on sequence homology, therefore it is more efficient than HDR even though it does not produce substitution of the mutated gene. HITI was modelled as a potential approach to insert a correct copy of *CFTR* cDNA within the endogenous locus even though it has not been experimentally validated yet [44]. An alternative targeted integration approach was achieved by the insertion of a *CFTR* super-exon (exons 11-23) targeting via ZFN the 5′ end of exon 11 in the *CFTR* locus in epithelial cell lines. While this strategy could potentially correct all the mutations located after exon 10, including the F508del, the complete phenotypic reversion was obtained only after antibiotic selection and in a small percentage of clones [45].

## 4. Gene Correction by Homology Directed Repair

Gene substitution is a very attractive procedure in gene therapy to replace a mutated sequence with a correct copy by exploiting the cellular HDR DNA repair pathway. HDR is very infrequent (1 out of 10^6^ events [22]) and thus is hardly exploitable in experimental setup and gene therapy. Key experiments demonstrated that an ectopic DNA sequence, the donor, bearing homology arms with the target site can substitute the endogenous sequence following DNA double strand break at the target site [16,23]. Sequence replacement occurs with significantly increased frequency at specific sites by using nucleases inducing DSBs [16,23]. These observations prompted the search for programmable nucleases to cleave specific sites in the genome where the repair substitution should occur. Genome editing technological advancement improved HDR also in cells that are normally refractory to gene replacements techniques [46]. Even though this editing strategy is still quite inefficient, it is so far the preferred choice to repair mutations. The first editing tool designed to target *CFTR* was a ZFN [47] applied by Harrison’s group in 2012 in cells derived from ΔF508 patients as a proof of concept for HDR gene correction [48]. This approach was further explored in iPS cells derived from patients using either ZFN [49], TALEN [50,51] or CRISPR-Cas9 [52].

CRISPR-Cas9 was proven valid to correct ΔF508 in advanced cellular models, such as intestinal organoids derived from patients, where CFTR channel function could be restored [53]. To compensate for low HDR efficiency in these studies, selection markers into the donor template, such as puromycin, were applied to enrich and isolate the corrected clones. To avoid clonal selection for detection and measurement of the substituted sequences by HDR, a sensitive sequencing technique was developed [54]. In vitro cellular enrichment, however, is not compatible with clinical applications. In order to enhance gene correction Porteus group exploited the intrinsic properties of AAV favoring HDR when used to deliver the donor template [55]. Without selection, from 30% to 40% of the mutated alleles were repaired in primary airway stem cells showing functional recovery of the CFTR channel. In this study the edited cells were embedded in FDA-approved porcine epithelial small intestinal submucosal membrane (pSIS) obtaining large number of CFTR expressing cells, potentially usable for transplantation in the upper-airway of CF patients [56]. These are encouraging data on the preserved nature of stem cells after genome editing in spite of p53 activation reported to occur in hematopoietic stem cells after DSB generated by programmable nucleases [57]. Electroporation is the preferred method for CRISPR-Cas9 delivery ex-vivo which allows transient expression of the nuclease and limited off-target activity [58]. Nevertheless, electroporation is not compatible for in vivo use, where either viral or non-viral tools remain an indispensable requirement, urging the development of specific systems of delivery for CF [59]. Despite the numerous attempts to correct the highly frequent ΔF508 deletion, the low efficiency of HDR prevents its broad use and translation to any clinical use. Development of molecular strategies enhancing HDR may provide in the future sufficient levels of gene substitution for its advancement to a therapeutic use [60].

## 5. Gene Correction by Non-Homologous End Joining

Clinical and preclinical works are testing programmable nucleases as DNA cleaving tools to inactivate genes involved in infectious diseases [27,28] or in dominant negative genetic pathologies [61]. Aside from disrupting an open reading frame, CRISPR-Cas genomic cleavage was also applied to restore genetic functions such as reinstating open reading frame in the dystrophin gene, mutated in Duchenne muscular dystrophy [62,63,64], or disruption of splicing regulatory regions to induce expression of the SMN2 gene in spinal muscular atrophy (SMA) mutated cells [65].

CRISPR-Cas mediated sequence cleavage was successfully used in CF to repair splicing mutations, accounting for about 10% of CF cases [66] where the production of aberrant mRNA transcripts impairs the expression of CFTR. Valuable models to set up gene correction strategies for these types of mutations are minigene models simulating the splicing defects [67,68,69]. Minigenes were used to prove that mutations 1811+1.6kbA>G, 3272-26A>G and 3849+10kbC>T located in introns following their cleavage with SpCas9 with gRNA pairs efficiently reversed the altered splicing [68]. This experimental design was further improved with AsCas12a to repair the 3272-26A>G and 3849+10kbC>T splicing aberration with a single gRNA, thus facilitating the efficiency and limiting the potential of non-specific (off-target) cleavages [69]. Both primary epithelial cells derived from patients and intestinal organoids were repaired in the 3272-26A>G and 3849+10kbC>T mutations with up to 90% efficiency and CFTR function recovery near wild-type levels for both mutations [69].

## 6. Correction of Point Mutation with Base Editors

DSB dependent editing by programmable nucleases activates DNA damage signaling pathway and may results in unpredicted rearrangements and translocations [70,71]. To avoid the potential pitfalls of DSB, a new class of Cas derived technologies were developed. CRISPR base editors are genome editing tools that insert single base modification into the target DNA sequence avoiding the formation of a DSB and without the requirement of a donor template [72]. Base editors are composed by a Cas nickase fused to a deaminase enzyme, either adenine or cytosine deaminases, that perform A to G or C to T base conversion, respectively, enabling four types of transition (A to G and C to T or G to A and T to C when targeted to the complementary strand) [72,73,74,75,76]. Based on the data that almost 60% of known diseases are caused by a point mutation, CRISPR base editors represent a powerful tool to study and correct these pathogenic variants [35]. The application of base editors is in principle limited exclusively by the presence of a compatible PAM (the sequence where Cas binds before the annealing of the complexed guide RNA) and an efficient method of delivery that is particularly demanding due to the large size of these tools. While the method of delivery remains a concern, in particular for in-vivo applications, the PAM limit will be very likely surpassed by the continued advancement of new Cas engineered variants with very little PAM requirements [77].

This technology was applied in CF by Clevers group to correct nonsense mutations in patients’ derived organoids carrying the R785X, W1282X or R553X mutations. Selected organoids showed A to G conversion in the expected positions with notably minimal efficiency (not more than 8% for any of the three tested mutations) [78]. Base editors delivered as chemically modified mRNA were tested in vitro to correct the most common CFTR nonsense mutation, W1282X. This study obtained efficient reversion of this point mutation and suggests a new method to deliver base editors [79].

## 7. Present and Future Gene Correction through Prime Editing

Prime editing is a CRISPR-based technology composed of a Cas9 nickase fused to an engineered reverse transcriptase (RT) and complexed with a prime editing guide RNA (pegRNA) [36]. The pegRNA differs from the regular gRNA by carrying a sequence that is reverse transcribed by the RT module to produce a donor DNA template. Based on the pegRNA design the outcome of prime editing is either an insertion, a deletion or a substitution with high level of precision. Therefore, this new technology potentially greatly expands the number of disease-causing mutations that can be corrected, including CF. As suggested in the original article, prime editing can be potentially applied to repair the most common cause of CF, by inserting three nucleotides in the mutated ΔF508 locus [36]. The complexed design of the prime editing guide RNAs was recently simplified by an ad hoc computational tool [80].

## 8. Modulating RNA in CF

Antisense oligonucleotides (ASOs) have been widely explored to therapeutically modulate gene expression by degradation of the target mRNA or by modulation of splicing elements for exon retention or skipping [81]. ASOs were applied in CF models to target aberrant donor and acceptor splice sites for the restoration of correct splicing events and the production of mature RNA and functional protein [82,83,84].

An alternative use of ASOs consisted in inserting the missing bases in 508 CFTR at the level of RNA transcripts in a ΔF508 cellular model. Even if a phenotypic reversion was measured, correction of the mRNA does not represent a stable modification and the mechanism of correction is not fully understood [85].

Instead of targeting *CFTR*, ASOs were used to block the epithelial sodium channel, ENaC, in mice models with CF-like symptoms [86]. The rationale behind this strategy derives from evidence that ENaC contributes to the disruption of the airway surface hydration mucus accumulation through increased Na^+^ absorption [87]. Indeed, ENaC was reported hyperactivated in cells carrying an altered CFTR function [87]. The aerosol delivered ASOs improved CF-like symptoms by restoration of mucociliary clearance and hydration of airway surface liquid. These results suggest that this is an attractive approach that can be used both as a monotherapy or in combination with other therapies to ameliorate CF conditions [86].

The ASO therapy is limited by the life-long dependency on repetitive treatments and adverse effects that still need to be addressed [81].

Another RNA-based approach to restore the proper amounts of CFTR transcripts was tested with the delivery of a lipid-based nanoparticle of chemically modified mRNA in patient-derived bronchial epithelial cells, showing increased membrane-localized CFTR [59]. The nasal application of the CFTR mRNA in CFTR knockout mice showed a proper chloride secretion in conductive airway epithelia up to two weeks from the treatment [59].

The Spliceosome mediated trans-splicing (SMaRT) technique was developed to repair the nascent mRNA by replacing part of the altered transcript with a correct exogenous mRNA [88]. This approach was proven to be valid in cellular models to reinstate correct transcripts from the mutated ΔF508 locus [89,90,91,92].

Phenotypic reversal using methods modulating CFTR mRNA by delivery or modification using ASOs or SMaRT offers the advantage of preventing potential genotoxicity generated by manipulation of the genome. However, RNA-based therapies transiently restore the function of CFTR, but are limited by requiring continuous lifetime treatments.

## 9. Genome Editing for the Development of CF Models

Experimental models resembling the CF phenotype, pathogenesis and symptoms are crucial for setup and evaluation of therapeutic strategies. Genome manipulation tools and the advancements of CRISPR-Cas technology gave a major impulse to the advancement of gene therapy solutions. These tools are also fundamental to advance gene therapy and drug development more in general by simplifying the production of new cellular and animal CF models [93,94]. Patient-derived cells are often used to test the efficacy of a treatment for a given mutation. However, considering the high number of *CFTR* variants associated with CF, it is very difficult to cover the entire mutation repertoire in homozygous cellular models. To overcome this limitation, isogenic models for different mutations were created using CRISPR-Cas9 mediated HDR in immortalized bronchial epithelial cells providing valuable experimental tools for CFTR functional assay [95].

CRISPR-Cas technology has been also applied to generate new CF animal models. A knockout sheep was generated by the disruption of the *CFTR* gene with CRISPR-Cas9 mediated NHEJ. CFTR^-/-^ sheep developed a severe disease similar to CF including pancreatic fibrosis, intestinal obstruction and the absence of vas deferens [96]. To specifically study the pathology caused by the most common ΔF508 mutation, rat models were generated exploiting the HDR pathway activated by CRISPR-Cas9 cleavage in correspondence with the phenylalanine 508 of the gene. Recombination with the donor DNA, containing the three nucleotides deletion, produced a homozygous rat model for the ΔF508. Compared with knockout rats, they showed a residual CFTR activity and a consequently milder CF phenotype [97].

A mouse carrying the G542X nonsense mutation in the *CFTR* locus was generated by CRISPR nucleases showing common manifestations of CF determined by the absence of CFTR ion channel, such as intestinal obstruction and reduced growth [98].

In conclusion, CRISPR genome editing is a very promising technology to generate new therapeutic strategies, as well as new valuable experimental tools to test therapies for a wide variety of CF causing mutations [93].

## 10. Conclusions and Future Directions

We have presented here emerging technologies and derived strategies as a path forward from conventional gene therapy based on delivery of therapeutic nucleic acids toward gene correction of *CFTR* mutations. Genome editing is at the forefront not just by offering novel therapeutic solutions but also for the development of experimental models, cellular and animal, essential in translational medicine. Although there is much to be done in particular in terms of tools for in-vivo delivery of nucleic acids and genome editing molecules, hurdles and limitations seem well-defined and manageable.

## Figures and Tables

**Table 1:** Genome editing techniques applied in CF models and main reported limitations.

Method	CFTR Defects	Experimental Model	Main Limitations
Gene complementation	all	experimental clinic	non endogenous promoter, possible loss of expression
HDR	all	iPS cells, organoids	low efficiency
HITI	all	not reported	DNA cleavage mediated
Super-exon	mutations located after the integration	epithelial cells	Delivery of the system
Directed NHEJ	splicing	minigene, epithelial cells and organoids	Off targets
Base editors	point mutations	organoids	In vivo delivery and low efficiency
Prime editing	all	not reported	In vivo delivery
Transcription regulators ASO	splicing	mice	requires continuous administration
SMaRT	all	cellular models	requires continuous administration
RNA treatments	all	mice	requires continuous administration
